# 63 Adjunctive Ketamine Reduces the Risk of Developing Chronic Opioid Use in Burn Patients

**DOI:** 10.1093/jbcr/irad045.037

**Published:** 2023-05-15

**Authors:** Dalton Amador, Isabel Obias, Kathleen Karam, Chris Soudah, Amy Arceneaux, Amina El Ayadi, Juquan Song, Georgiy Golovko, Steven Wolf

**Affiliations:** The University of Texas Medical Branch, Galveston, Texas; The University of Texas Medical Branch, Galveston, Texas; The University of Texas Medical Branch, Galveston, Texas; The University of Texas Medical Branch, Galveston, Texas; The University of Texas Medical Branch, Galveston, Texas; The University of Texas Medical Branch, Galveston, Texas; The University of Texas Medical Branch, Galveston, Texas; The University of Texas Medical Branch, Galveston, Texas; The University of Texas Medical Branch, Galveston, Texas; The University of Texas Medical Branch, Galveston, Texas; The University of Texas Medical Branch, Galveston, Texas; The University of Texas Medical Branch, Galveston, Texas; The University of Texas Medical Branch, Galveston, Texas; The University of Texas Medical Branch, Galveston, Texas; The University of Texas Medical Branch, Galveston, Texas; The University of Texas Medical Branch, Galveston, Texas; The University of Texas Medical Branch, Galveston, Texas; The University of Texas Medical Branch, Galveston, Texas; The University of Texas Medical Branch, Galveston, Texas; The University of Texas Medical Branch, Galveston, Texas; The University of Texas Medical Branch, Galveston, Texas; The University of Texas Medical Branch, Galveston, Texas; The University of Texas Medical Branch, Galveston, Texas; The University of Texas Medical Branch, Galveston, Texas; The University of Texas Medical Branch, Galveston, Texas; The University of Texas Medical Branch, Galveston, Texas; The University of Texas Medical Branch, Galveston, Texas; The University of Texas Medical Branch, Galveston, Texas; The University of Texas Medical Branch, Galveston, Texas; The University of Texas Medical Branch, Galveston, Texas; The University of Texas Medical Branch, Galveston, Texas; The University of Texas Medical Branch, Galveston, Texas; The University of Texas Medical Branch, Galveston, Texas; The University of Texas Medical Branch, Galveston, Texas; The University of Texas Medical Branch, Galveston, Texas; The University of Texas Medical Branch, Galveston, Texas; The University of Texas Medical Branch, Galveston, Texas; The University of Texas Medical Branch, Galveston, Texas; The University of Texas Medical Branch, Galveston, Texas; The University of Texas Medical Branch, Galveston, Texas; The University of Texas Medical Branch, Galveston, Texas; The University of Texas Medical Branch, Galveston, Texas; The University of Texas Medical Branch, Galveston, Texas; The University of Texas Medical Branch, Galveston, Texas; The University of Texas Medical Branch, Galveston, Texas; The University of Texas Medical Branch, Galveston, Texas; The University of Texas Medical Branch, Galveston, Texas; The University of Texas Medical Branch, Galveston, Texas; The University of Texas Medical Branch, Galveston, Texas; The University of Texas Medical Branch, Galveston, Texas; The University of Texas Medical Branch, Galveston, Texas; The University of Texas Medical Branch, Galveston, Texas; The University of Texas Medical Branch, Galveston, Texas; The University of Texas Medical Branch, Galveston, Texas; The University of Texas Medical Branch, Galveston, Texas; The University of Texas Medical Branch, Galveston, Texas; The University of Texas Medical Branch, Galveston, Texas; The University of Texas Medical Branch, Galveston, Texas; The University of Texas Medical Branch, Galveston, Texas; The University of Texas Medical Branch, Galveston, Texas; The University of Texas Medical Branch, Galveston, Texas; The University of Texas Medical Branch, Galveston, Texas; The University of Texas Medical Branch, Galveston, Texas; The University of Texas Medical Branch, Galveston, Texas; The University of Texas Medical Branch, Galveston, Texas; The University of Texas Medical Branch, Galveston, Texas; The University of Texas Medical Branch, Galveston, Texas; The University of Texas Medical Branch, Galveston, Texas; The University of Texas Medical Branch, Galveston, Texas; The University of Texas Medical Branch, Galveston, Texas; The University of Texas Medical Branch, Galveston, Texas; The University of Texas Medical Branch, Galveston, Texas; The University of Texas Medical Branch, Galveston, Texas; The University of Texas Medical Branch, Galveston, Texas; The University of Texas Medical Branch, Galveston, Texas; The University of Texas Medical Branch, Galveston, Texas; The University of Texas Medical Branch, Galveston, Texas; The University of Texas Medical Branch, Galveston, Texas; The University of Texas Medical Branch, Galveston, Texas; The University of Texas Medical Branch, Galveston, Texas; The University of Texas Medical Branch, Galveston, Texas

## Abstract

**Introduction:**

Opioid use for perioperative pain management in burn patients is associated with a high risk of developing chronic opioid use. Ketamine is commonly used as an analgesic adjunct due to its opioid-sparing effects. The purpose of this study is to determine whether analgesia with adjunctive ketamine reduces the risk of developing chronic opioid use in burn patients.

**Methods:**

Burn patients who underwent burn-related surgeries were identified using relevant ICD-10 Codes. Data were collected using the TriNetX Research Network, a national research database providing de-identified patient medical records.

Burn patients undergoing surgical procedures were identified based on their use of opioids alone or in combination with ketamine as adjunctive analgesia. Both groups excluded patients with a prior history of opioid-related and psychiatric disorders to control for potential pre-existing conditions affecting the study outcomes. Outcome analysis included continued opioid use and related psychiatric disorders, including nicotine dependence and anxiety, up to one year after burn injury. Risk ratios for each outcome were then generated using a measure of association analysis through TriNetX.

**Results:**

We identified 107,270 patients across 51 health care organizations who underwent burn-related surgeries within one month after burn injury. Excluded from the study were 3,296 (3.07%) patients who had a prior history of opioid-related and psychiatric disorders. The remaining 103,974 patients were sorted into respective groups that received opioids with ketamine (n = 8,328) and without ketamine (n = 51,413). The two cohorts were balanced using (1:1) propensity score matching for age, sex, race, ethnicity, and burn surface area.

Patients without ketamine had a higher risk of continuing opioid use (risk ratio [RR], 1.14, p < 0.001), developing nicotine dependence ([RR], 1.32, p < 0.001), and developing anxiety disorders ([RR], 1.21, p = 0.007) at least one year after burn injury.

**Conclusions:**

Burn patients whose pain management included adjunctive ketamine ceased opioid use sooner and demonstrated a lesser risk of developing various psychiatric disorders associated with chronic opioid use.

**Applicability of Research to Practice:**

This study supports the use of adjunctive ketamine as part of a multimodal approach to pain management in burn patients in order to minimize the risk of chronic opioid use.

